# Health Alliances: a new way of working internationally

**DOI:** 10.1186/s12913-025-12353-0

**Published:** 2025-02-13

**Authors:** Omolade Allen, Ketsina Ewusie, Amy Waghorne, Ged Byrne, Lucie Byrne-Davis, Jo Hart

**Affiliations:** 1 Division of Medical Education, School of Medical Sciences, The University of Manchester, Manchester, UK; 2 Health Education England, Manchester, UK

**Keywords:** Health partnership, Global health, International collaboration, Health Alliances

## Abstract

**Background:**

International collaborations have been pivotal in global health delivery, particularly in addressing challenges like tuberculosis. Recently, there has been a shift towards more focused alliances designed for targeted collaboration. This study examines health alliances as a distinct model of international partnership in global health, exploring their operational approaches, key activities, encountered challenges, and potential contributions.

**Methods:**

A qualitative approach was utilised, in which ten semi-structured interviews were conducted with individuals involved in various health alliances. These were transcribed, anonymised, and analysed to identify common themes.

**Results:**

Four main themes were generated, revealing that health alliances operate through collaborative efforts pooled from diverse stakeholders, including governments, and academic institutions. Serving as platforms for open communication, these alliances facilitate the exchange of expertise, resources, and best practices.

The study highlights key activities undertaken by health alliances, including research, capacity building, policy engagement, and resource mobilisation. These activities strengthen health systems, generate new knowledge, and mobilise financial and technical resources.

**Conclusion:**

This research demonstrates the potential of health alliances as effective models for future collaborations in global health. By addressing the challenges of fragmentation, lack of coordination, and limited focus on capacity building and evaluation, health alliances enhance the impact of global health initiatives. The findings contribute to a better understanding of health alliances and could inform the development of future alliances.

## Introduction

Global Health Partnerships are pivotal in addressing worldwide health delivery disparities. A World Health Organisation (WHO) study across 133 countries, representing 87 per cent of the global population, highlighted income disparities as a key factor contributing to weak healthcare systems in low- and middle-income countries (LMICs) [1]. Health systems in high-income countries (HICs) are well-equipped, whereas LMICs often lack human capacity, technology, and infrastructure, leading to poor health outcomes [2, 3]. These differences led to the formation of international partnerships for knowledge-sharing and coordinated positive health delivery involving collaborations among non-governmental organisations (NGOs), international and national health institutions, civil society, and the private sector [4, 5]. Examples of partnerships include the Global Fund to Fight AIDS and the United Nations Secretary General's Every Woman and Every Child Initiative [6].

Health partnerships, therefore, have become indispensable in bridging health inequality gaps. A study of 219 partnerships found 72 per cent lasted over five years and 60 per cent involved bi-directional visits [7]. Whilst this study focused on virtual partnerships, it presents a snapshot of the scope of such collaborations.

On capacity building, for instance, The THET Partnership for Global Health has been supporting overseas health workers since 1989. It has since trained over 50,000 health workers across thirty-four countries in Africa, the Middle East and Asia [8]. Despite progress, a need for more focused collaboration persists [1, 9]. Hence, health alliances emerged as a responsive approach to equitable partnerships [10].

Emerging health alliances, such as the Myanmar UK Health Alliance (MUKHA), and the Uganda UK Health Alliance (UUKHA), focus on country-specific need [10]. This study, therefore, focuses on exploring the perspectives of those involved in Health Alliances to examine their operational activities and assess how effectively they could foster a renewed and results-oriented paradigm for an efficient global health delivery system.

### What are global health partnerships (GHPs)?

A global health partnership (GHP) describes collaborative efforts between organisations, governments, and other stakeholders, primarily from the global north, to address global health challenges in LMICs [11, 12]. It involves sharing resources, expertise, and knowledge to improve health outcomes, strengthen healthcare systems, and address cross-border health concerns [5, 11]. GHPs can take various forms and focus on a wide range of health concerns, including infectious diseases, non-communicable diseases, maternal and child health, healthcare infrastructure, and capacity building [12–15] These partnerships often involve international organisations, such as the WHO, governments, NGOs, academic institutions, charities, and private sector entities [16].

### Aims of GHPs

The primary objectives of GHPs vary, often focusing on capacity building to strengthen healthcare systems by upskilling professionals in LMICs [12, 17]. For instance, the Midwives Emergency Skills Training programme (MEST) illustrates this through collaboration between Canadian and Tanzanian midwives, working in rural areas to improve emergency obstetrics training [15]. These partnerships also drive research, innovation, policy influence, and resource mobilisation [18]. The 2006 Project GLOBE in New York exemplifies evidence-based medical education development for primary care physicians in LMICs [19, 20]. Through strategic advocacy and policy engagement, these partnerships aim to influence health policies and priorities at national and international levels [21, 22]. Lastly, global health partnerships are instrumental in mobilising financial and technical resources to ensure the sustainability and effectiveness of interventions [23, 24].

### Challenges faced by GHPs

Despite their positive efforts, GHPs have encountered notable challenges. Historically, these collaborations have suffered from fragmentation and a lack of coordination among stakeholders, resulting in duplicated efforts, inefficient resource utilisation, and a lack of synergy [9, 22, 23]. A glaring example is evident in "The Global Fund", which targeted Sierra Leone and Uganda, facing poor communication between stakeholders and diverting attention from in-country priorities [9, 23].

Additionally, this fragmentation has led to inadequate resource allocation, encompassing financial, human, and infrastructural aspects [9]. Given limited funding and resource disparities between countries, implementing effective health programmes across LMICs is challenging [9, 17]. Historically, HICs dominated collaboration terms, limiting LMICs' participation in decision-making processes, thus undermining the sustainability and relevance of initiatives [25–28]. This power imbalance perpetuates resource inequities and fuels debates surrounding the need for the "decolonisation of healthcare" [25, 29, 30].

Another critical issue is the limited emphasis on capacity building and knowledge transfer [31]. Many collaborations focused primarily on short-term interventions over long-term healthcare system development [11, 31]. This has hindered the sustainability and autonomy of healthcare systems in LMICs, further limiting their ability to address local health challenges effectively. Monitoring and evaluation mechanisms have also been insufficient, hindering the assessment of intervention impact [17, 32, 33]. A review of 74 GHPs found that most of these partnerships failed to express 'specific and measurable' impeding performance monitoring, making it challenging to learn from failures and improve future collaborations [32].

### Emergence of health alliances

Health alliances have emerged as a new approach to international collaboration, aiming to bolster the effectiveness of GHPs in improving global health outcomes while addressing coordination challenges [13, 28]. The current alliances are the Kenya-UK Health Alliance (KUKHA), MUKHA, UUKHA and the Zambia-UK Health Workforce Alliance (ZUKHWA) [10].

ZUKHWA, established in 2009 at Guys and St Thomas's NHS Foundation Trust, London, represents a combined network of Zambian and UK-based healthcare stakeholders, including the Zambian Ministry of Health and Anglia Ruskin University [34, 35]. ZUKHWA supports the Zambian government’s two main objectives: provision of health education opportunities and health system development [34, 36].

UUKHA, established in 2013 by the Ugandan Ministry of Health and NHS Health Education England, aims to deliver UK-derived health programmes in Uganda through robust collaboration, resource-sharing, and professional development [10, 35]. UUKHA's initiatives include the Human Resources for Health (HRH) programme, encouraging professional exchange between the UK and Uganda, and the B.R.I.D.G.E programme, promoting collaborative research [10, 35]. Similarly, MUKHA, founded in 2013, enhances collaboration between the Myanmar Ministry of Health and Sports and UK partners, focusing on medical and nursing education, General Practitioner training, emergency preparedness, digital health, mental health support, and advocacy [10, 37].

KUKHA, formalised in 2020 through a memorandum of understanding (MoU), signifies a three-decade effort by Kenya and the UK to strengthen healthcare outcomes across both nations [38]. These alliances exemplify a promising shift towards more effective and mutually beneficial international health collaborations.

### How do health alliances address the issues of GHPs

The term "Health alliance", refers to a collaboration between a diverse set of stakeholders in HICs with organised healthcare systems like the UK, and a supported country of interest. These alliances encompass entities such as the UK government, NGOs, academic institutions, the private sector, and civil society organisations, all striving to improve health outcomes, including healthcare infrastructure, reducing disease transmission, and increasing life expectancy [11, 39]. They also emphasise health equity in LMICs grappling with disparities in healthcare access due to factors including income inequality and low economic development. While initiatives such as the Gavi Vaccine Alliance contribute to health inclusivity [40], GHPs have been criticised for cultural insensitivity, task duplication and power imbalances [31].

Both Health Alliances and GHPs strive to address intricate health challenges in LMICs by leveraging stakeholders' strengths [3]. Health alliances, however, enhance GHPs by fostering cooperation, sharing expertise, and pooling resources through open communication and trust-building, effectively addressing the problems of task repetition and fragmentation [19].

Health Alliances stand out by prioritising consistent communication channels within their networks, fostering cooperation between beneficiaries, and mitigating duplication of efforts [9–11, 39]. This approach simplifies collaborative efforts, ultimately leading to efficient health interventions. Moreover, Health Alliances adopt a holistic multisectoral approach, acknowledging the complex web of socio-economic, environmental, and political factors influencing health outcomes. This ensures that health interventions address the root causes of inequities, such as poverty and limited access to healthcare and education. Furthermore, these alliances empower marginalised communities, ensuring their voices are heard in health policy and practice.

Health alliances also foster innovation and knowledge exchange, creating a platform for ideas and best practices to circulate, leading to the development of novel health solutions [19]. By promoting innovation and knowledge sharing, health alliances can help build a culture of learning and continuous improvement in the health sector. This can lead to improved health outcomes, more efficient use of resources, and a more responsive and resilient health system.

However, despite their potential, there is a lack of comprehensive understanding regarding health alliances' structure, functioning, and the factors contributing to their success. Furthermore, limited research has been conducted exploring the perspectives and experiences of key stakeholders involved in these alliances. This knowledge gap hinders the ability to leverage the full potential of health alliances. Thus, this study explores existing health alliances to provide insights into their structure and functioning. By uncovering lessons learned from these alliances, this study will contribute to a better understanding of the challenges faced by health alliances and inform the development and implementation of future alliances, ultimately enhancing their impact on addressing healthcare challenges in LMICs.

## Method

### Research objectives

This study aims to understand how health alliances work, their activities, difficulties encountered, and how they can be further strengthened.

The specific objectives are to:

1. Assess the operational methods and strategies health alliances employ in global health collaboration.
2. Identify the activities and interventions undertaken by health alliances to address global health challenges.
3. Investigate the challenges and potential of health alliances as effective models for future global health collaborations.

### Study design

The present study utilises a qualitative research approach involving semi-structured interviews. The semi-structured approach was chosen because it allows the participants to provide open-ended responses [41], leading to the collection of more comprehensive information. The aim was to obtain rich and detailed accounts of participants' perspectives, experiences and thoughts about working with or for a health alliance.

### Data collection

**Fig. 1:**
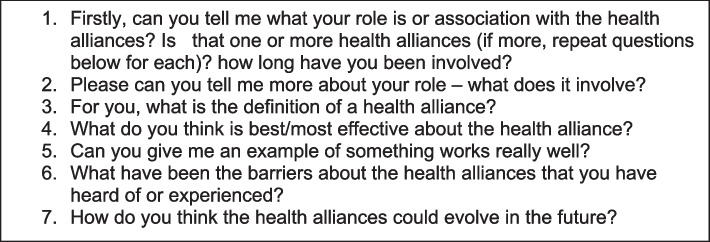
List of interview guide questions used for data collection

Participants were recruited via email from those who have worked with a health alliance, been involved in their development, oversight, or part of a partnership that has worked with a health alliance on a convenience sample. MUKHA, UUKHA, KUKHA and ZUKHWA were included. Ten Interviews were conducted virtually over Microsoft Teams by the researcher, who had no previous relationships with study participants. The researcher conducted the interviews in their home office with no one else present. However, who else was present on the participants' side is unknown. The interviews were semi-structured with follow-up questions based on participants' responses. Interview guide questions (Fig. 1) explored participants' roles within health alliances, their opinions on what works well within the alliances, possible barriers they have faced, and how they thought the alliances could develop in the future. Interviews were audio-recorded and transcribed using Microsoft Teams, at a convenient time and date for both the participant and the researcher. Once transcribed, audio recordings were deleted. The interviews lasted between 30–45 min.

### Data analysis

The audio recordings of the interviews were transcribed verbatim within one week of the interview and anonymised one week after the interview. Data analysis of the anonymised transcripts was conducted using a thematic analysis approach. The analysis enabled the identification and exploration of patterns and themes in the data collected from the interviews [42]. The researcher began by reading the transcripts multiple times to familiarise themselves with the data and identify initial patterns. The researcher then began to code the transcripts in NVIVO 12 version 12.5.0.815. The initial codes were then arranged and categorised to form potential themes [42]. The research team met virtually to discuss findings from the data and how they aligned with the potential themes. These themes were reviewed and modified by the researcher based on the discussion. The themes were adjusted until the research team were satisfied.

### Characteristics of participants

There were 10 participants involved in this study, and their corresponding involvement in a health alliance has been anonymised below, replacing country names with numbers (see Table 1).

**Table 1:** Table of participant characteristics

*Participant Number*	*Gender*	*Associated health alliance/s*	*UK- based or international*
*Participant 1*	Female	Country 3	Internationally based
*Participant 2*	Male	Country 1, 2, 3	UK based
*Participant 3*	Male	Country 4	UK based
*Participant 4*	Male	Country 2	Internationally based
*Participant 5*	Male	Country 4	UK based
*Participant 6*	Male	Country 1, 2, 3	UK based
*Participant 7*	Male	N/A	UK based
*Participant 8*	Male	Country 4	UK based
*Participant 9*	Male	Country 1, 2, 3, 4	UK based
*Participant 10*	Male	Country 4	UK based

## Results

### Thematic analysis

**Fig. 2:**
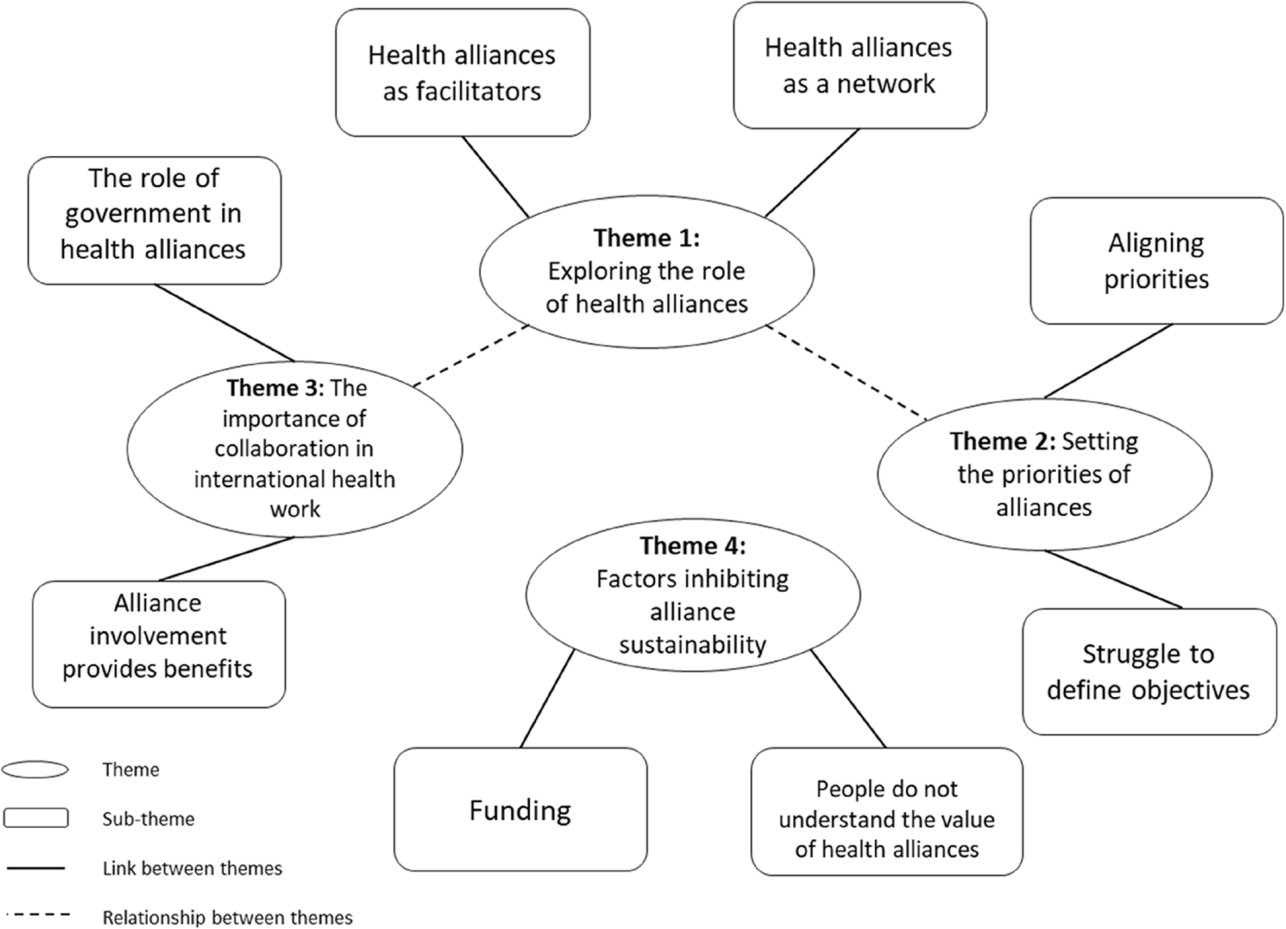
Mind map presenting main themes and sub-themes

Four main themes were identified. These are: exploring the role of health alliances, setting the priorities of health alliances, the importance of collaboration in international health work and finally, factors inhibiting alliance sustainability and recommendations for future collaborations. These main themes have sub-themes presented below (see Fig. 2).

### Theme 1: exploring the role of health alliances

#### Health alliances as facilitators

There was an overall consensus that health alliances took on a more facilitatory role. Participants highlighted that alliances primarily aim to encourage their members to increase their collaborative efforts, ensuring coordination and minimising duplication of work. This emphasises the importance of alliances in fostering synergy among its members and promoting collective action towards shared goals. These responses accentuated the role of health alliances in facilitating effective collaboration, harmonising efforts, and maximising the collective potential of diverse stakeholders in addressing global health challenges.

"The Alliance itself is to encourage its members to do more work, to coordinate it, to make sure that there isn't a lot of duplication going on." (P8)

"Within the Alliance, the work is to make sure that all of those different projects that are being done are all done in parallel, they're all organised, make the biggest possible change without setting up conflicts, without setting up duplications, and so on." (P3).

#### Health alliances as a network

Health alliances were regarded as a network bringing people together and fostering collaboration among diverse institutions and actors ranging from governments to smaller health partnerships, NGOs, universities, and hospitals. Health alliances were also described as a community of institutions working towards a common goal, particularly in strengthening health systems and advancing health services in multiple countries. This exemplifies how health alliances serve as networks that facilitate collective efforts towards shared objectives.

"So, I think it's about bringing people together." (P2)

"It is the community or a network of institutions working together towards a common goal, and especially in, you know, health systems strengthening and the advancement of the health services in both countries." (P1).

"I suppose from my perspective a health alliance … is really a, I would say, it's a program of work which is designed around the connection of many different actors who are working in the space of health systems strengthening or health workforce strengthening in a particular country." (P2).

"It's giving you that linkage, because on Country 4's side it links into government, it links into The Ministry of Health, it links into the county governments, as well as the hospitals and universities…" (P3).

### Theme 2: Setting the priorities of health alliances

#### Aligning priorities

Health alliances focus on aligning priorities. Participants emphasised the importance of ensuring that their work aligns with existing national health strategic plans or workforce development plans. This demonstrates the commitment of health alliances to align their activities with the broader national health agenda, thereby maximising their impact and relevance whilst also tackling the power imbalances categorically seen in traditional health partnerships.

"So, where we work, we try to ensure that whatever we're doing finds reference in, for instance, there might be a National Health strategic plan or a National Health workforce development plan or something like that." (P2).

Aligning priorities within health alliances is a collaborative process. Participants described gathering key stakeholders and engaging in discussions to identify and narrow down priorities. For example, one interviewee explained how they gathered stakeholders and discussed priorities in Country 3, eventually narrowing down to five key focus areas: medical education, nursing education, GP training, pathology services, and digital health information. Health alliances also engaged with governments to address priority areas, allowing them to shape government initiatives and interventions to align with these areas.

"We managed to gather and invite all the key stakeholders… and together with the UK partners, we were able to discuss what were the priorities at that time for the country… after intensive exchange and discussion, we managed to narrow it down to five priorities where both countries can focus and work meaningfully." (P1).

"The alliance right now is able to negotiate government programs. Right now, it's handling the three core government programs that are focused on supporting the healthcare system. One on human resource, the other on investments and finance, and the third one on research and development." (P4).

#### The struggle to define objectives

Health alliances struggle to define clear objectives. Participants highlighted the challenges and complexities associated with determining the precise objectives of these alliances. This suggests that the process of defining the objectives of health alliances requires further exploration and understanding.

"I think there are so many unknowns that involve alliances, which we will have to put a bit more work onto it so that going forward, we can better define what alliances and where alliance sit within the government diplomacy work." (P1).

Furthermore, interviewees noted that the collaboration of multiple groups within health alliances contributes to the difficulty in establishing clear objectives. The complexity and diversity of stakeholders involved in health alliances can lead to a vague understanding of the alliance's objectives, prolonging the process of establishing clear goals and further highlighting the ongoing challenge health alliances face in formulating precise and well-defined objectives.

"One of the barriers or difficulties with the alliances is how long it has taken them to really get a sense of what clear objectives are for the alliance. And I think that comes from them being a collaboration of many different groups." (P2).

"That work of developing clear objectives of what the alliance was trying to accomplish was also a little bit hazy for quite a long time." (P2)

"The barrier is trying to find a common endpoint. You know everybody likes to talk about alliances, but trying to define what are these outcomes, what you know, and how do you measure them" (P5).

### Theme 3: The importance of collaboration in international health work

#### The role of government in health alliances

There is a crucial role of government in health alliances. Participants emphasised the significance of government recognition and support in facilitating the work of the alliances. When the government recognises and supports the work of health alliances, it can significantly ease the partnership's operations and enable smoother implementation of initiatives by opening doors to resources and assistance.

"It could make things so much easier for that partner if the government recognises that work." (P2)

"If the government has said OK, we like what you're doing, we want to support it, then they might, for instance, help by providing that partnership with assistance with immigration." (P2).

Several respondents highlighted the benefits of having access to government officials and senior government representatives. This access opened doors for interactions and meetings with key stakeholders, enhancing the alliance's ability to navigate the health landscape effectively.

"We've been very fortunate we've had access to government and senior government, and they've been able to open doors for us to interactions and meeting with people." (P3)

Participants expressed the importance of government engagement and financial support in sustaining health alliances. They emphasised the need for the UK government, which had previously provided funds, to continue supporting these alliances financially. While acknowledging the voluntary contributions from alliance members, they underscored that government involvement and financial contributions were necessary for health alliances’ long-term success and stability.

"I'm hoping that the UK government, which has provided funds in the past, will look again, and start providing a certain amount of money together with, as I say, contribution from our members." (P8).

However, the need for a balanced approach to the involvement of governments was also recognised. The responses highlighted the importance of genuine partnerships between governments, who bear the primary responsibility for the health of their nations, and organisations that voluntarily participate in health alliances. It was noted that alliances structured solely as government bodies, driven solely by government priorities, may fail to adapt and sustain themselves over time. Genuine partnerships, characterised by shared responsibility and voluntary participation, were viewed as the key to the effective functioning of health alliances.

"It's also important that governments are involved…but if they end up by being kind of government bodies and they, they will fail simply because the government's come and go and they have their own priorities. But if they're genuine partnerships between governments…and organisations that are willing to participate on a voluntary basis, then I think they'll work very well." (P8).

#### Alliance involvement provides benefits

The interview responses revealed a key theme surrounding the numerous benefits of being involved in health alliances. Participants emphasised the unique opportunities that alliance membership provides, particularly in accessing senior government officials on both sides of the alliance. Being a member of the alliance grants organisations access to high-level government representatives, offering a level of engagement that would not have been possible otherwise. This access to senior government officials enhances the visibility and influence of participating organisations, opening doors for collaboration, and amplifying the impact of their work.

"If you're a member of the alliance, you're likely to get to see senior people, in the governments on both sides." (P8)

Participants highlighted the significant time and resource-saving advantages that alliance involvement brings. Organisations gain access to comprehensive information and resources from relevant sources by being part of the alliance. This access streamlines operations, ensuring that organisations have the necessary guidelines and information readily available, thereby saving valuable time and resources that would have been spent searching for or acquiring such information independently.

"It will definitely save time and resources… we have all the information that any organization needs Regarding the Ministry of Health, their policy guidelines from their side, as well as any other organizations who are working in Country 3 from UK side." (P1).

Additionally, alliance involvement fosters more excellent connectivity and collaboration within the country. Participants emphasised that alliances could connect many in-country actors, amplify their work, and enhance their effectiveness. These institutional linkages and collaborative networks become more resilient and effective due to the presence and support of the alliance.

"They can also open pathways in terms of connecting them to other facilities or other actors in country to help amplify the work that they're doing and to help make it more effective" (P2).

Furthermore, interviewees highlighted the potential for bidirectional learning within health alliances. They argued that alliances could serve as conduits for knowledge exchange, allowing for the sharing of experiences, best practices, and lessons learned between different stakeholders. This bidirectional learning fosters a culture of continuous improvement and innovation within the alliance, enhancing the collective knowledge base and overall effectiveness of its members.

"I think an argument needs to be made, perhaps a bit more strongly as well, that alliances could be a conduit for bidirectional learning as well." (P2)

"This August 15 medical students from the UK will be going to Country 4 to do student electives" (P5)

Alliance involvement also provides opportunities for organisations, especially charities, to showcase their work to a wider audience. Being an alliance member enables organisations to gain more visibility and credibility, significantly impacting their fundraising efforts and supporting their mission.

"But by being members of the alliance, they are now able to. Show that if you show their work to wider audience, which maybe helps them encourage helps them with the fundraising and also gives them more credibility." (P8).

### Theme 4: Factors inhibiting alliance sustainability

#### Funding

One significant theme that emerged from the interviews was the challenge of securing sufficient funding for the sustainability of health alliances. Participants highlighted the struggle faced by alliances in securing funding due to their primary role of coordination and facilitation. This highlights the difficulty in attracting funding for activities not directly tied to the programmes’s implementation or service delivery.

"At the moment, the alliance will probably struggle to secure funding because what the alliance does is mainly coordination, facilitating." (P1)

The different funding mechanisms and practices in different contexts further compounded the funding issue. Participants emphasised the contrasting financial systems between the UK and partner countries. For instance, one participant mentioned the difference in payment procedures between UK universities and the Country 4 system. This disparity in financial systems poses a challenge to the financial sustainability of alliances, especially when upfront funding is required.

"In the UK, we're very used to…spend some money and then get paid 3 months in arrears…But in Country 4, that simply does not work. You actually have to feed money upfront." (P3).

The lack of clarity and uncertainty surrounding funding sources and mechanisms emerged as a significant barrier to alliance sustainability. Participants expressed the need for clear funding mechanisms and specific projects within the alliance's research and teaching agenda to attract funding. However, the lack of clarity limits the scope and potential of alliances. Funding streams may change, requiring the alliance to adapt and explore different funding sources.

"I suppose money probably is a barrier… because the funding of the alliances and how they work isn't entirely clear." (P7)

Moreover, participants noted that alliances often rely on voluntary efforts, with varying levels of available time and resources among alliance members. The limited funding restricts the activities that alliances can undertake and their ability to scale up their efforts. Additionally, participants mentioned that alliances currently receive direction and funding from other parts of the healthcare system, such as NHS England and Health Education England, indicating a lack of dedicated funding for alliance operations.

#### People do not understand the value of health alliances

One prominent theme that emerged from the interviews was the challenge of demonstrating the value and importance of health alliances, which posed a barrier to their sustainability. Participants expressed concerns about the perception that individual organisations could implement projects and activities without the involvement of alliances. This suggests a lack of recognition regarding the crucial role of alliances in mitigating duplication of work and promoting coordination among diverse stakeholders.

"Then people could argue that…we can go directly to the Ministry of Health without involving the alliance and we can do it our own way." (P1)

The struggle to argue the value of alliances was linked to funding challenges and the need for wider recognition of the importance of alliance work. Participants highlighted the funding aspect as a significant barrier, emphasising that people currently do not fully recognise the importance of alliances. This lack of recognition and understanding of the value of alliances hindered their ability to secure sustainable funding and support.

"The main barrier is sort of the funding aspect and the fact that people don't really recognise as of now the importance of the alliance's work." (P1)

The ability to provide sufficient benefits to individual alliance members was also raised as a consideration for sustainability. Participants questioned whether the alliances delivered enough value to justify membership and potential subscription fees. This further emphasises the importance of demonstrating alliances' tangible benefits and value proposition to ensure their continued support and engagement.

"Are we providing enough benefit for the individual members…to be part of the alliance?" (P2)

To overcome these challenges, participants suggested the need to successfully argue for the value of collective efforts and the unique benefits of the alliance model. They highlighted the importance of showcasing the advantages of collaborative work and the quality of the health partnership model. This indicates the importance of effectively communicating the broader benefits and outcomes achieved through alliance collaboration, in attracting support from funding sources that appreciate the unique advantages of the alliance approach.

"If they can successfully argue…that the collective effort…is of greater quality…then I think they can work to try to find those kinds of sources of funding that believe in that model." (P2).

## Discussion

The findings of this study provide meaningful insights into the operational methods, key activities, challenges, and potential of health alliances to function as effective models for collaboration in global health. These insights are discussed in relation to the three key objectives of this study. The first is the assessment of the operational methods and strategies employed by health alliances in international collaboration for global health.

The interviews revealed that health alliances take on a facilitatory role, encouraging collaborative and coordinated efforts among diverse stakeholders, including governments, non-governmental organisations, academic institutions, and the private sector. By leveraging the strengths and resources of participating organisations, health alliances address the issues of fragmentation and lack of coordination [9], that have been observed in traditional global health partnerships. The network created through alliances facilitates efficient collaboration, leading to more effective and targeted interventions for addressing complex health challenges.

The second objective, which was to identify the key activities and interventions undertaken by health alliances to address global health challenges, was recognised through the interviews that highlighted the various priorities the health alliances such as MUKHA and UUKHA focussed on. These included human resource development, investments and finance, and research and innovation. Across the conducted interviews, it was discovered that human resource development efforts focused on strengthening healthcare systems by enhancing the skills and knowledge of healthcare professionals in LMICs. Research and innovation activities aim to generate new knowledge and evidence-based interventions to improve health outcomes globally. Health alliances also engage with governments to influence health policies and priorities at national and international levels.

Finally, this project investigated the challenges and potential of health alliances as effective models for future collaborations in global health. The findings suggest that health alliances have the potential to address the challenges faced by traditional global health partnerships. By promoting coordination, information sharing, and efficient resource allocation, alliances enhance the impact of global health initiatives. They also facilitate the development of partnerships and networks that can lead to sustainable solutions for health challenges. However, further work is needed to define clearer objectives of health alliances and to secure effective funding modalities.

### Comparison with existing knowledge

Health alliances as a model are effective in rectifying the issues of traditional GHPs. The existing knowledge about GHPs, their objectives, challenges, and limitations. Traditional GHPs and health alliances share a common objective of addressing global health concerns, particularly in LMICs, and both strive to strengthen health systems in these areas [10]. However, health alliances distinguish themselves by emphasising and encouraging the coordination and collaboration of various stakeholders involved in the health sector [10]. Participants expressed that by bringing together governments, NGOs, charities, academic institutions, and other organisations, health alliances aim to improve the efficacy and sustainability of their work. This coordinated approach recognises the need to leverage multiple stakeholders' expertise, resources, and perspectives in building more robust health systems in LMICs [9, 22, 23].

Fragmentation, power imbalances, inadequate capacity building, and limited focus on monitoring and evaluation have often characterised traditional partnerships [9, 25–28]. These challenges have hindered the effectiveness and sustainability of interventions. On the other hand, health alliances offer a new approach that addresses these issues by promoting coordination, cooperation, and efficient resource allocation.

Fragmentation of traditional health partnerships leads to the duplication of efforts, as seen in the duplication of planning arrangements of The Global Fund [23]. Participants in this study highlighted the role of health alliances in actively minimising duplication by ensuring efficient coordination of efforts. They emphasised the alliances' role of encouraging its members to collaborate and coordinate their work, thereby avoiding unnecessary duplication. By fostering collaboration and alignment among stakeholders, health alliances aim to ensure that different initiatives are conducted in parallel and organised to maximise their collective impact without creating conflicts or duplications.

Health alliances can address the issue of power imbalances in traditional global health partnerships by aligning with in-country priorities. For example, the global bioethics discourse serves as an illustrative example of this imbalance. Significant disparity exists in access to the published ethics literature, making it exceedingly challenging for bioethicists from LMICs to learn from and contribute to the global bioethics body of knowledge [25]. Participants highlighted that health alliances, through their collaborative and inclusive approach, strive to bridge this gap by actively engaging with in-country stakeholders and aligning their efforts with national health priorities. By acknowledging and valuing local professionals' and communities' expertise and perspectives, health alliances foster a more equitable distribution of power and decision-making, leading to a more inclusive global health discourse. This approach addresses the issue of power imbalances, and ensures that interventions and policies are rooted in the specific context and needs of LMICs, promoting sustainability and effectiveness in achieving health outcomes.

Overall, the findings indicate that health alliances can maximise efficacy through combined efforts. Participants emphasised that health alliances align their work with national health plans and engage in collaborative processes to identify and streamline priority areas. This approach ensures that the alliance's efforts are focused and targeted, maximising their impact on the healthcare system. By aligning priorities, health alliances can effectively contribute to advancing key areas such as medical education, healthcare workforce development, and digital health, among others.

### Limitations

Firstly, the study is limited to participants who have worked with a health alliance, been involved in their development, have oversight, or were part of a partnership that has worked with a health alliance. This may limit the range of experiences captured. To capture a wider range of experiences, future studies could expand the participant pool to include individuals who have not only worked directly with a health alliance, but also those who have indirect involvement or have been impacted by alliance activities. This can provide a more comprehensive understanding of the perspectives and experiences related to health alliances.

Secondly, the sample size is relatively small, limiting the generalisability of the findings. A larger sample size could increase the generalisability of the findings. Future research can aim to include a larger and more diverse group of participants, ensuring representation from various stakeholder groups and geographic regions. This will help to validate the results and provide a more comprehensive picture of the challenges and potential of health alliances.

Finally, the study is limited to a qualitative approach using semi-structured interviews, which may limit the depth and breadth of the data collected. Combining interviews with surveys or other quantitative measures can help gather quantitative data on participants' perceptions, attitudes, or outcomes related to health alliances. This would provide a more comprehensive and nuanced understanding of the topic.

### Areas of further work

The issue of unclear objective setting, observed in traditional global health partnerships [9], seems to have translated into health alliances, as a key theme identified was the difficulty in objective setting. The findings indicate that health alliances encounter difficulties in defining clear objectives, which can be attributed to the complexity of their collaborative nature and the diverse range of stakeholders involved. The process of clarifying objectives requires additional effort and time to ensure a shared understanding and direction within the alliance. This signifies that more work is needed to suitably set specific and measurable objectives to ensure the work that alliances facilitate is effective. Addressing this struggle to define objectives is crucial for enhancing the effectiveness and impact of health alliances in addressing global health challenges.

The importance of adequate funding is highlighted in traditional global health partnerships and health alliances. There is a need for sustained financial resources to support the initiatives and interventions carried out by these collaborative models. Insufficient funding has been a significant challenge in global health partnerships, leading to limitations in the scope and scale of their activities [16, 33]. Participants revealed that health alliances face similar funding constraints, hindering their potential for impact and sustainability. Adequate funding is crucial for implementing long-term strategies, conducting research and innovation, strengthening healthcare systems, and addressing health inequities effectively [9, 15, 43]. Therefore, addressing the funding gap is an essential area of further work for health alliances. This includes advocating for increased investment in global health, exploring innovative funding mechanisms, and forging partnerships with philanthropic organisations and donors. By securing adequate financial resources, health alliances can increase their capacity to expand their reach, implement impactful interventions, and ultimately contribute to achieving global health objectives sustainably and equitably.

## Conclusions

This study aimed to assess the operational mechanisms and strategies employed by health alliances in international collaboration for global health, identify and analyse the key activities and interventions undertaken by health alliances to address global health challenges, and evaluate the potential of health alliances as effective models for future collaborations in the field of global health. Through qualitative analysis of interviews, it can be concluded that health alliances are effective as a new model of global health collaboration. Comparing the findings with existing knowledge, this research contributes to understanding the operational mechanisms, challenges, and potential of health alliances in global health collaborations. These insights can inform the development and implementation of future alliances, enhance their impact, and foster effective collaborations to address complex global health challenges.

## Data Availability

The datasets generated and analysed during the current study are not publicly available due to ethical restrictions, but are available from the corresponding author on reasonable request.

## Abbreviations

GHP: Global Health Partnership
WHO: World Health Organisation
NGO: Non-governmental Organisations
LMIC: Low- and Middle-income Countries
HIC: High Income Countries
MUKHA: Myanmar UK Health Alliance
UUKHA: Uganda UK Health Alliance
ZUKHWA: Zambia-UK Health Workforce Alliance
KUKHA: The Kenya-UK Health Alliance
